# Treatment of Digital Ischemia with Liposomal Bupivacaine

**DOI:** 10.1155/2014/853243

**Published:** 2014-02-05

**Authors:** José Raul Soberón, Scott F. Duncan, W. Charles Sternbergh

**Affiliations:** ^1^Department of Anesthesiology, Ochsner Clinic Foundation, 1514 Jefferson Highway, New Orleans, LA 70121, USA; ^2^Department of Orthopedic Surgery, Ochsner Clinic Foundation, 1514 Jefferson Highway, New Orleans, LA 70121, USA; ^3^Vascular and Endovascular Surgery, Ochsner Clinic Foundation, 1514 Jefferson Highway, New Orleans, LA 70121, USA

## Abstract

*Objective*. This report describes a case in which the off-label use of liposomal bupivacaine (Exparel) in a peripheral nerve block resulted in marked improvement of a patient's vasoocclusive symptoms. The vasodilating and analgesic properties of liposomal bupivacaine in patients with ischemic symptoms are unknown, but our clinical experience suggests a role in the management of patients suffering from vasoocclusive disease. *Case Report*. A 45-year-old African American female was admitted to the hospital with severe digital ischemic pain. She was not a candidate for any vascular surgical or procedural interventions. Two continuous supraclavicular nerve blocks were placed with modest clinical improvement. These effects were also short-lived, with the benefits resolving after the discontinuation of the peripheral nerve blocks. She continued to report severe pain and was on multiple anticoagulant medications, so a decision was made to perform an axillary nerve block using liposomal bupivacaine (Exparel) given the compressibility of the site as well as the superficial nature of the target structures. *Conclusions*. This case report describes the successful off-label usage of liposomal bupivacaine (Exparel) in a patient with digital ischemia. Liposomal bupivacaine (Exparel) is currently FDA approved only for wound infiltration use at this time.

## 1. Introduction

Chemical sympathectomy from peripheral nerve blockade is well known and especially beneficial in vascular surgery [1]. Limited data are available regarding chemical sympathectomy in patients with digital ischemic pain and vasculopathy. Patients with these conditions, especially those with ischemic pain secondary to autoimmune disorders, benefit from chemical and surgical sympathectomy [2, 3]. The benefits of surgical sympathectomy often persist for years after the surgical procedure [3].

Exparel is a liposomal bupivacaine formulation that is currently FDA approved only for wound infiltration use. While this novel anesthetic is most commonly used during bunion and hemorrhoid surgery, it is also used in a number of other colorectal and orthopedic procedures and is gaining popularity in other surgical specialties [4, 5]. Its vasodilatory properties are unknown at this time.

## 2. Case Report

Written informed consent was obtained from the patient after a lengthy discussion of the known and potential risks and benefits of the block procedure, and all questions were answered. Witnessed verbal consent was obtained from the patient prior to the preparation of this case report. Additionally, the Ochsner Clinic Foundation Institutional Review Board was contacted and determined that IRB approval was not necessary for the submission of this case report.

A 45-year-old African American female presented with a two-week history of severe pain in the 4th and 5th digits of her right hand. Her past medical history was significant for poorly controlled type I diabetes, hypertension, peripheral vascular disease, and opiate dependence. Imaging studies of the extremity revealed diminished flow and occlusion of the distal ulnar artery. Her physical exam revealed cyanosis of the 4th and 5th digits with limited hand movement secondary to discomfort.

The vascular surgery team postulated that her ulnar artery was injured during attempts at intravascular catheter placement during a recent admission for diabetic ketoacidosis. After angiography, the vascular surgeons determined that she was not a candidate for direct revascularization with intra-arterial thrombolysis or operative intervention. The patient was not offered an amputation by the surgical team at this time because the ischemic regions of her hand had not fully demarcated and to allow the possibility of healing if nonsurgical interventions were successful. Additionally, she initially declined an amputation when the possibility of one was discussed during her hospitalization. A heparin infusion and oral aspirin were initiated for anticoagulation therapy. Anesthesiology was then contacted to place a nerve block to assist with pain control and provide a chemical sympathectomy. A supraclavicular nerve catheter was placed under Ultrasound guidance, and her pain and clinical symptoms modestly improved over the next two days. Her nerve catheter was removed and she was discharged home. Aspirin and clopidogrel were prescribed for outpatient use.

However, she was readmitted to the hospital two days later complaining of continued pain in her hand. Clinical examination showed that her condition had regressed to what it was during her initial presentation, with increased cyanosis when compared to prior to discharge. In addition to aspirin and clopidogrel, enoxaparin and coumadin were added to her anticoagulation regimen, and the anesthesia team was consulted to place a second nerve block.

A supraclavicular nerve catheter was placed but became dislodged less than 48 hours after its insertion. The patient was scheduled for a repeat block procedure with the expectation that she would be discharged home soon afterwards if her pain and symptoms improved. She was determined not to be a suitable candidate for an outpatient perineural catheter.

An ultrasound-guided Axillary block was performed using 1.3% liposomal bupivacaine (Exparel), with 3 mL injected incrementally around the musculocutaneous, radial, median, and ulnar nerves. A total of 12 mL of liposomal bupivacaine was used, corresponding to a total dose of 159.6 mg. A repeat supraclavicular catheter was not performed because of the patient's anticoagulated state and the possibility of hospital discharge if her symptoms improved. She was monitored in the block area 30 minutes after the procedure and placed on continuous telemetry monitoring for the remainder of her hospital stay.

She continued to report high pain scores in spite of appearing more comfortable following the procedure. The next day, the vascular surgeon noted an improvement in her physical examination, with normal color in the 5th digit and up to the dorsal aspect of the distal 1/3 and plantar to the proximal 1/3 of the 4th digit. She also had increased grip strength and hand movement.

These clinical improvements were markedly better than what she experienced with the previous supraclavicular blocks, and she was discharged home. She did not experience any signs or symptoms of local anesthetic toxicity at any point during her hospitalization, and she denied them on daily telephone followup for five days.

**Figure 1:**
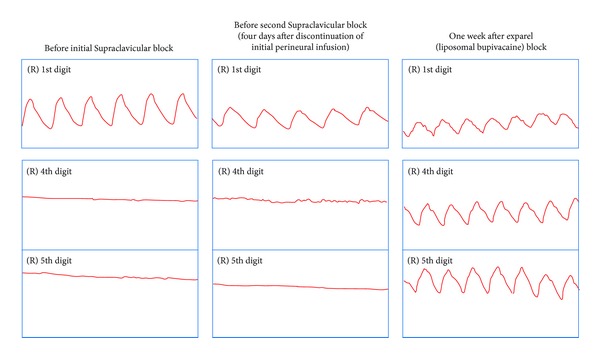
Results from photoplethysmography (PPG) studies obtained throughout the patient's hospitalizations. Of particular interest is the sustained blood flow seen in the 4th and 5th digits one week after her liposomal bupivacaine (Exparel) block. The 1st digit was nonischemic and included as a control.

Photoplethysmography (PPG) flow studies provide a qualitative measure of digital arterial perfusion by measuring the digital artery pulsatility. Progressive reduction in digital arterial flow creates damping of the waveform. A “flatline” suggests very poor arterial flow. These PPG studies were performed during her hospital admissions, and the results are illustrated in Figure 1.

She demonstrated no pulsatility in the affected 4th and 5th digits prior to treatment, consistent with the clinical presentation. Although the patient's pain and clinical symptoms improved while receiving a perineural infusion of local anesthetics, these benefits did not persist after discontinuation of her peripheral nerve catheter, and no improvements in digital blood flow were seen in PPG studies. Moreover, increased flow in her 4th and 5th digits was observed on the PPG studies one week after the liposomal bupivacaine block.

Unfortunately, the patient's ischemic vasculopathy worsened over the subsequent six weeks, and her 4th finger became gangrenous. She was scheduled for a 4th digit amputation and surgical sympathectomy of the 4th and 5th digits as an outpatient. During her surgical procedure, however, it was found that her common digital arteries were clotted. A surgical sympathectomy was nonetheless performed even though the efficacy of doing this procedure in vessels that are already clotted is unknown. The patient underwent an amputation at the level of the proximal interphalangeal joint to remove the gangrenous digit. Her amputation site healed without complication, and she has had no other ischemic issues with the remaining digits. On followup, she reported better pain control and use of the hand compared to prior to surgery.

## 3. Discussion

Our decision to use an off-label medication was based on multiple factors, with patient safety being at the forefront. Our indication for a block in this scenario, unlike most of our routine cases, was not postoperative pain control, avoidance of a general anesthetic, or minimization of opioid-related side effects, but a last-effort attempt at preserving the patient's digits.

We believe that our clinical decisions, albeit controversial, were based on careful consideration of the benefits and risks, specifically improving blood flow in an attempt to salvage at least part of a limb against the possibility of nerve injury, local anesthetic toxicity, or other potential (currently unknown) risks associated with liposomal bupivacaine. Given her history of opioid dependence, her pain was difficult to control with oral and intravenous analgesics. At the time of the liposomal bupivacaine block, the patient was on multiple anticoagulant medications, each acting on a different facet of the coagulation process. An axillary block was deemed safer than a repeat supraclavicular block on an anticoagulated patient, because of ease of block performance and compressibility of the site should bleeding occur. Furthermore, the block was performed by a Fellowship trained regional anesthesiologist (J. R. Soberón) with extensive experience in Ultrasound-guided peripheral nerve blocks. A strict in-plane technique was used to visualize the needle at all times to avoid vascular trespass or intraneural injection. Meticulous and frequent aspiration were negative for blood during the block procedure. Avoiding a block altogether would have likely led to worsening ischemia and the loss of the 5th digit, as well as a prolonged hospitalization for pain control purposes.

Exparel is a liposomal bupivacaine formulation that is currently FDA approved only for wound infiltration use, although FDA trials for perineural use approval are currently underway. Its vasodilatory properties are unknown at this time.

Patients with vaso-occlusive disorders benefit from peripheral nerve blocks because of their vasodilating and analgesic effects. In addition to chemical sympathectomy, surgical sympathectomy may be attempted to increase blood flow to the digits [2, 3]. The most common surgical approach involves identifying the common digital arteries and removing the adventitia from the vessels over a 1- to 2- centimeter segment. This removal of the adventitia disrupts the zone where the nerves lie, allowing for subsequent vasodilation and potentially increased blood flow.

It is unclear if improved control of the patient's medical issues, as well as increased adherence to therapy and medical recommendations, would have yielded a better outcome. Our patient was spared amputation of the 5th digit, which was also ischemic and cyanotic during her presentation. Our PPG results and clinical experience suggest that liposomal bupivacaine may increase blood flow and assist with pain control in patients with digital ischemia. Further research needs to be performed to determine the safety and potential role of liposomal bupivacaine in peripheral nerve block use, particularly with regards to analgesic and vasodilating effects in patients with ischemic symptoms.

## References

[1] Malinzak EB, Gan TJ (2009). Regional anesthesia for vascular access surgery. *Anesthesia and Analgesia*.

[2] Greengrass RA, Feinglass NG, Murray PM, Trigg SD (2003). Continuous regional anesthesia before surgical peripheral sympathectomy in a patient with severe digital necrosis associated with Raynaud’s phenomenon and scleroderma. *Regional Anesthesia and Pain Medicine*.

[3] Murata K, Omokawa S, Kobata Y, Tanaka Y, Yajima H, Tamai S (2012). Long-term follow-up of periarterial sympathectomy for chronic digital ischaemia. *J Hand Surg Eur*.

[4] Candiotti K (2012). Liposomal bupivacaine: an innovative nonopioid local analgesic for the management of postsurgical pain. *Pharmacotherapy*.

[5] Pacira Pharmaceuticals Inc (2011). *EXPAREL (Bupivacaine Liposome Injectable Suspension) Prescribing Information*.

